# What are the broader impacts and value from a randomised controlled trial conducted in six public hospital antenatal clinics in Australia? An impact assessment using the Framework to Assess the Impact from Translational health research

**DOI:** 10.1136/bmjopen-2023-082795

**Published:** 2025-03-26

**Authors:** Elissa Elvidge, Vanessa E Murphy, Melanie Rao, Peter G Gibson, Karen McLaughlin, Annelies Robijn, Megan E Jensen, Leonie Kaye Callaway, John Attia, Michael Hensley, Warwick Giles, Michael Peek, Helen Barrett, Sean Seeho, Joerg Mattes, Alistair Abbott, Andrew Bisits, Kirsten McCaffery, Paul B Colditz, Andrew Searles, Shanthi Ann Ramanathan

**Affiliations:** 1 School of Medicine and Public Health, College of Health, Medicine and Wellbeing, University of Newcastle, Callaghan, New South Wales, Australia; 2 Asthma and Breathing Research Program, Hunter Medical Research Institute, New Lambton Heights, New South Wales, Australia; 3 Health Research Economics, Hunter Medical Research Institute, New Lambton Heights, New South Wales, Australia; 4 Department of Respiratory and Sleep Medicine, John Hunter Hospital, New Lambton Heights, New South Wales, Australia; 5 School of Nursing and Midwifery, College of Health, Medicine and Wellbeing, University of Newcastle, Newcastle, New South Wales, Australia; 6 Obstetric Medicine, Royal Brisbane and Women’s Hospital, Brisbane, Queensland, Australia; 7 School of Medicine, University of Queensland, Brisbane, Queensland, Australia; 8 Specialty of Obstetrics, Gynaecology and Neonatology, Sydney Medical School Northern, University of Sydney, Sydney, New South Wales, Australia; 9 Australian National University Medical School, The Australian National University, Canberra, Australian Capital Territory, Australia; 10 Royal Hospital for Women Randwick, Randwick, New South Wales, Australia; 11 Paediatric Respiratory and Sleep Medicine Department, John Hunter Children’s Hospital, New Lambton Heights, New South Wales, Australia; 12 Nepean Hospital, Kingswood, New South Wales, Australia; 13 School of Public Health, University of Sydney, Sydney, New South Wales, Australia

**Keywords:** Asthma, Maternal medicine, HEALTH ECONOMICS, Clinical Trial, Primary Health Care

## Abstract

**Abstract:**

**Objectives:**

The Breathing for Life Trial (BLT) was a multicentre randomised controlled trial testing the hypothesis that a fractional exhaled nitric oxide-based intervention to guide asthma therapy in pregnancy improves perinatal outcomes. While BLT was negative based on selected outcomes, the conduct of the trial over 7 years showed potential for assessing the broader research impacts and returns on investment in BLT. The aim of this study was to retrospectively assess and report on the impact and value of BLT to show accountability for the research investment in what was deemed a ‘negative’ trial.

**Methods:**

The Framework to Assess the Impact from Translational health research (FAIT) was selected as the preferred method. FAIT combines three validated methods, including a modified Payback framework, an economic analysis of return on investment and a narrative account of the impact generated from the research. Data collection was done via document analysis of BLT administrative and research records and review of relevant websites/databases.

**Results:**

BLT delivered a return on investment of $6.7 million in leveraged grants, fellowships and consultancies and conservatively returned $2.44 for every dollar invested. The research trained and upskilled 18 midwives and obstetricians in evidence-based asthma management in pregnancy and improved research capability of six PhD students. Specialised equipment purchased by BLT is now being repurposed to undertake other research in regional Australia, saving further research investment. Of the 1200 mothers who were part of BLT, 508 now have written asthma plans, 268 had a clinically significant improvement in their asthma control score and the proportion who improved their asthma plan knowledge increased by 58 percentage points from 12 to 70%.

**Conclusion:**

This case example in the developing field of impact assessment illustrates how researchers can use evidence to demonstrate and report more broadly on the impact of and returns on research investment in a clinical trial.

**Trial registration number:**

ACTRN12613000202763; Post results.

**STRENGTHS AND LIMITATIONS OF THIS STUDY**

- A key strength of this work was the combination of three validated methods for assessing the impact of research: quantitative impact metrics, a cost-consequence analysis and a narrative.
- This comprehensive assessment methodology ensures the results have the potential to appeal to a wide audience, including researchers, policymakers and research funders.
- The retrospective application of Framework to Assess the Impact from Translational health research meant the impact assessment was necessarily conservative, and other impacts could potentially have been captured had an impact assessment been planned from the beginning.
- Limited funding meant no opportunity for primary data collection that would have evidenced the benefit and value to midwives, obstetricians and general practitioners involved in the trial.

## Introduction

 Every year Australia spends $10 billion on health and medical research, which is approximately 4% of all spending on health (total health expenditure in 2021–2022 was $241 billion).^1^ Of this expenditure, approximately 10% or $1.1 billion is spent on clinical trials activity.^2^ Clinical trials are important, providing essential evidence for more effective and lifesaving therapies and identifying ineffective and unnecessary interventions. They also help find ways to detect diseases early when they are more treatable. Clinical trials advance medical knowledge, which leads to better health outcomes for patients, and hospitals that conduct clinical trials tend to provide better care and have lower mortality rates.^3^

Despite this large investment of public monies and the global benefits that clinical trials can bring, very little is known about the benefits that result from individual clinical trials, especially when they have a negative result. With the impact of COVID and the rising cost of healthcare, there is an added imperative for health and medical researchers to be accountable for the spending of public monies. In Australia and globally, there is a growing demand for more accountability in public spending across all sectors, including health and medical research.^4^ Despite this, impact assessment beyond academic outputs such as peer-reviewed publication citations or field-weighted citation indexes is still not standard practice in many countries.^5^ The value of negative trials is often underestimated in terms of the new knowledge and benefits they generate over and above an often necessarily narrow set of outcomes to the exclusion of other potentially meaningful outcomes.

One such example is the Breathing for Life Trial (BLT), a multicentre randomised controlled trial (RCT) conducted between 2013 and 2019 that tested the hypothesis that better control of asthma in pregnancy and/or the prevention of asthma exacerbations in pregnancy could lead to improved outcomes for the baby at birth,^6^ and may be a primary prevention strategy for asthma in children at high risk of developing asthma.^7^ The trial tested the effectiveness of an intervention that used the level of eosinophilic lung inflammation (measured as fractional exhaled nitric oxide (FeNO)) to adjust asthma medication dose in pregnancy and asthma symptoms to determine the need for long-acting beta-agonist. The full protocol has been published^6^ as have the main trial results^8^ and other related data.^9 10^ BLT showed no differences between the usual care control group and the FeNO-based management intervention group in any of the perinatal outcomes examined.

Given the significant investment of public funds in this trial, the research team were keen to be as transparent and accountable as possible for the investment and assess the impact and value of BLT using a broader societal lens.

With support from the Health Economics and Impact team at the Hunter Medical Research Institute, we undertook a retrospective impact assessment of the BLT. The objective was to assess and evidence the broader impacts and benefits from the trial and give visibility to its value and return on the research investment.

## Methods

There are a plethora of impact assessment frameworks available internationally, including three systematic reviews of these frameworks, models and applications,^11^,^13^ but none have been applied to clinical trials and published.

FAIT was selected as the preferred framework due to its multidimensional lens on impact, its ability to be retrospectively applied and the opportunity to trial its feasibility for application to a clinical trial. Given there are no impact frameworks designed specifically for clinical trials, we believed the multidimensional feature of FAIT was the best approach for the purpose of conducting an impact assessment of the BLT.

**Figure 1:**
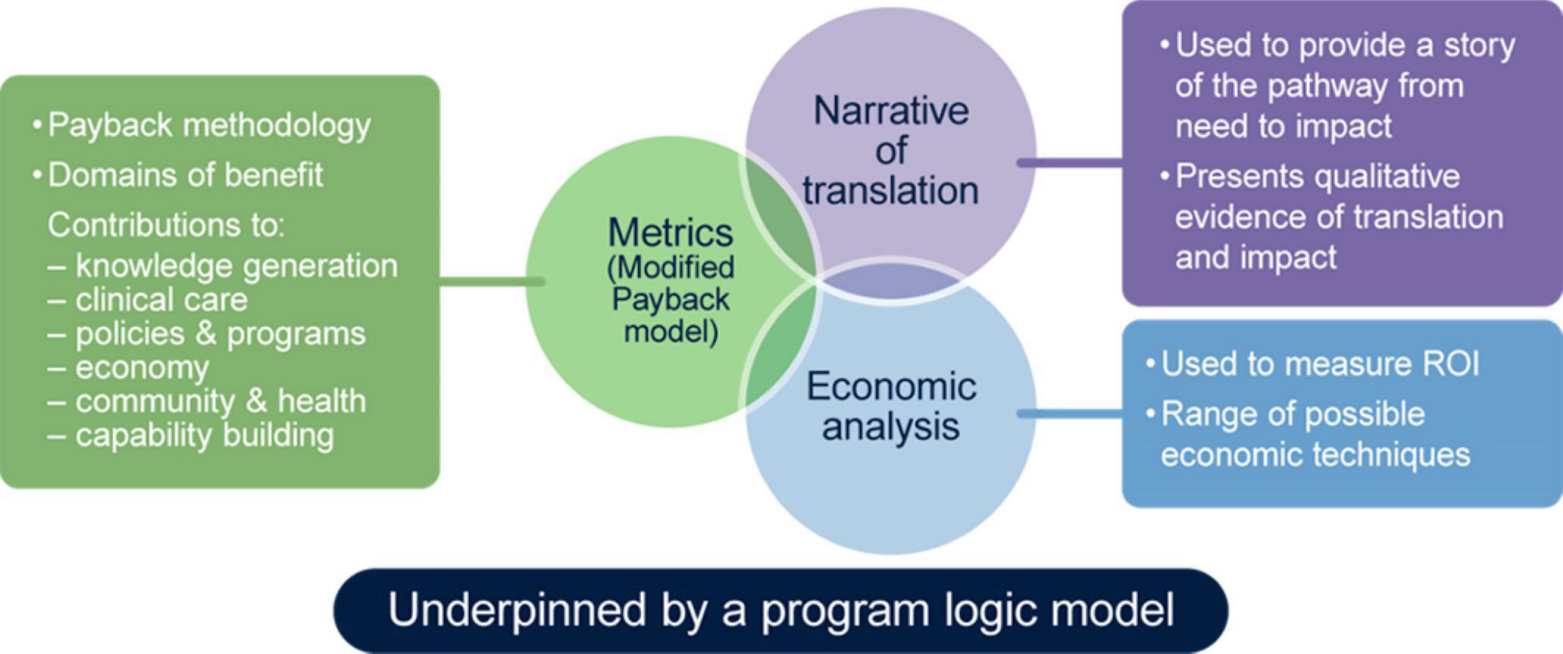
Graphical representation of the Framework to Assess the Impact from Translational health research, which incorporates three methods of impact assessment (quantified metrics grouped within the Payback domains of benefit, an economic analysis of the return on investment (ROI) and a narrative account of the research and benefits from the end user perspective).

FAIT is a hybrid of three proven methodologies for measuring research impact: quantified metrics, economic analysis and narratives of the process by which research translates and generates impact. Details about the development of FAIT can be found in the seminal FAIT paper.^14^ Figure 1 presents a pictorial representation of FAIT. A description of each phase as it was applied to BLT is given below.

### Program Logic Model

**Figure 2:**
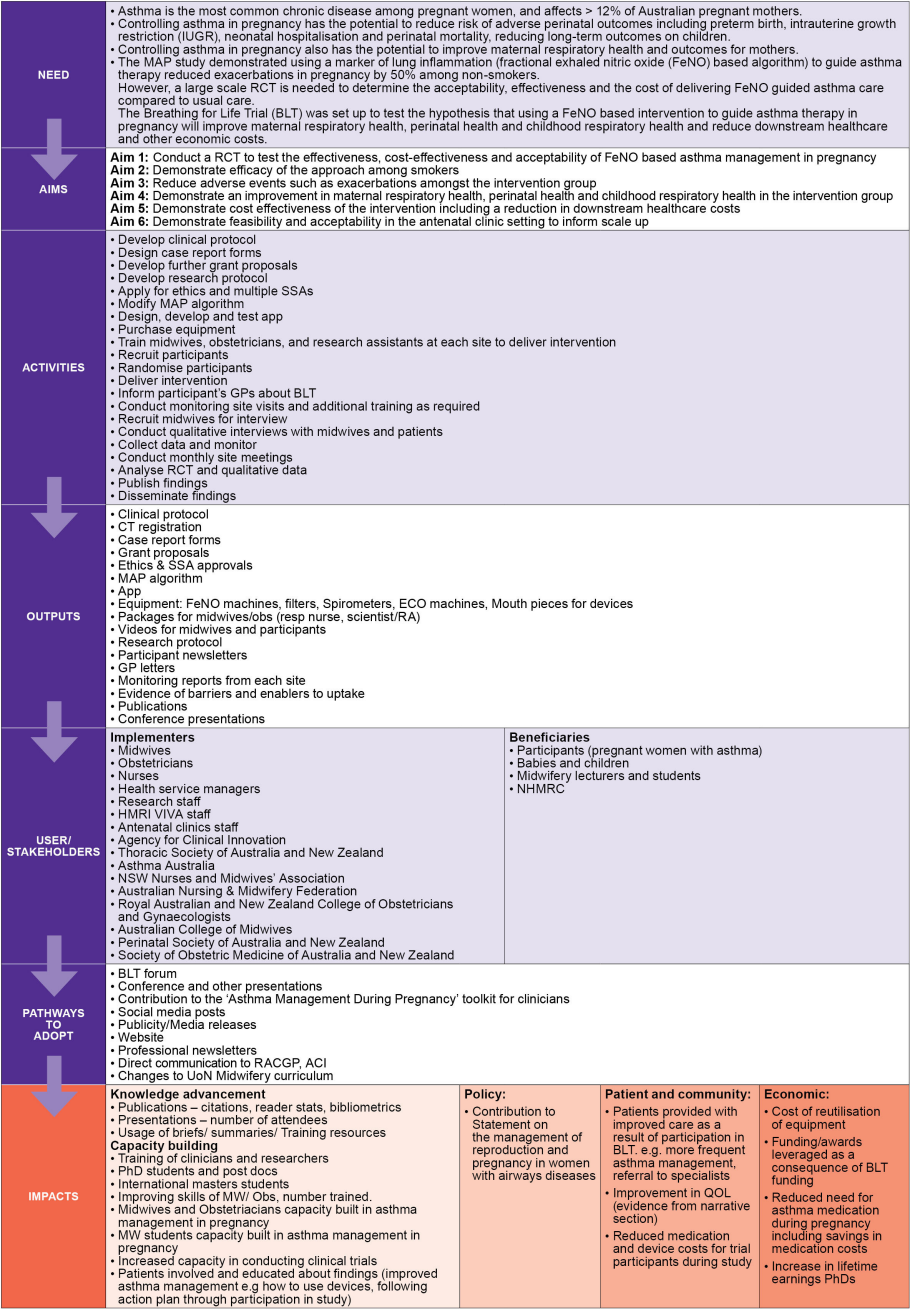
Customised Program Logic Model that was developed retrospectively to document the pathway to impact for the Breathing for Life Trial from the need for the research to the aspirational impacts. The program logic underpins the application of the Framework to Assess the Impact from Translational health research. ACI, NSW Agency for Clinical Innovation; GP, general practitioner; HMRI VIVA, Hunter Medical Research Institute, Viruses, Immunity, Vaccines and Asthma Program; MAP, Managing Asthma in Pregnancy Study; MW, midwifery; NHMRC, National Health and Medical Research Council; QOL, quality of life; RACGP, Royal Australian College of General Practitioners; RCT, randomised controlled trial; SSA, site specific governance approvals.

The three FAIT methods are underpinned by program logic modelling—a process that maps a pathway describing how a program is intended to have impact by linking aims, activities, outputs and impacts. Given this was a retrospective application, the Program Logic Model for BLT was developed by applying the key components to the existing pathway for BLT and used to retrospectively link the BLT needs, aims and activities to potential impacts within domains of benefit. The Program Logic Model was developed by the BLT team with support from impact practitioners (figure 2).

### Payback

Once the Program Logic Model was established, the Payback framework^14 15^ was used to identify the impact from BLT by selecting appropriate quantifiable impact metrics in each of the relevant domains. This was a modification on the original Payback framework that captures impacts using qualitative statements. An iterative process resulted in the selection of Payback metrics specific to BLT. These included more generalised metrics available from metric databanks. These were grouped under the following Payback domains: knowledge advancement, capacity and capability building, policy, community and economic impacts.

### Economic analyses

The economic analysis was designed to understand the return on investment in BLT. A cost-consequence analysis (CCA) was selected as the relevant and pragmatic method based on the availability of data and the lack of economic evidence generated as part of the trial. A CCA presents an array of consequences and costs in a disaggregated form.^16^ A CCA is intended to present a transparent account of costs and consequences across many different dimensions, allowing decision-makers to see clearly what types of information are included and omitted, and to make their own decision about the expected value of a program. Within the BLT application, the CCA involved monetary valuation of consequences wherever possible, leaving other consequences expressed in their natural units of measurements. The Payback table was used to record all the consequences of BLT using quantified metrics, leaving only the monetisable impacts to be included in the CCA results.

#### Costs

This study reports ‘economic costs’, which measure all resources used for BLT in monetary terms. A bottom-up approach was used to capture all resources expended on BLT from the perspectives of the research programme, clinicians and patients. All resources were identified, valued, aggregated and presented in monetary units so that the value of different resources can be aggregated and compared. The research grants were used to capture a bulk of the research costs. These included 100% of the main study grant from the Australian National Health and Medical Research Council, half the value of a career fellowship grant for the chief investigator, other top-up infrastructure and equipment funding from the University of Newcastle and other philanthropic organisations. Opportunity costs were also calculated for all meeting participation and non-paid contributions from other investigators which included senior staff specialists, nurses, midwives and several academic researchers and professional staff. Their time was provided as ‘in-kind’ contributions from their respective organisations. Wage rates were obtained from the published Australian National University rates,^17^ the NSW Health staff awards^18^ and converted to 2022 dollars,^19^ and each member of the team was mapped to their appropriate wage rate. The cost of overheads (electricity, water, security, building maintenance, etc) and oncosts (superannuation, leave, etc) were added to labour costs at a rate of 27.5% for overheads and 20.5% for oncosts. More than 90% of the 58 meetings were via teleconference, so no travel costs were included. The same method was used to value the opportunity costs for attendance at all training events of which there were 18. These attendees were mainly senior staff specialists, nurses, midwives and occasionally one additional academic researcher whose time was not paid for by the grant. Implementation costs were also estimated for participants based on their time for attendance at study visits. The average Australian wage from the Australian Bureau of Statistics was used to value their time^20^ and converted to 2022 values using the latest Australian National Accounts.^19^ Their time per visit was based on an estimation of the average time, and sensitivity analysis (SA) was used to account for variation in visit times. All costs were expressed in 2022 Australian dollars. Travel time and costs were not included in the implementation costs as details of these were not captured for the study.

#### Consequences

Consequences were focused on four main areas: (1) funding leveraged from BLT for further research in this field; (2) medication savings for patients given their medications were funded by BLT for the duration of the trial; (3) savings in repurposed specialised equipment that did not need to be purchased for subsequent research and (4) lifetime increase in earnings from PhD completions associated with BLT. The consequences included were limited to those that were monetisable. Consequences not readily monetisable are presented in their natural units and discussed within the Payback metrics (table 1). Attribution for the leveraged funding was determined on a scale of 0% (BLT did not contribute at all to the leveraged funding) to 100% (the funding would not have been leveraged without BLT) and values converted to 2022 values. The medication costs saved were obtained from the published 2022 prices on the Chemist Warehouse website (https://www.chemistwarehouse.com.au) for the four main drugs used by pregnant patients: Ventolin, 100 μg; Pulmicort, 100 μg; Symbicort 100/6 and Symbicort 400/12. The use of the market cost rather than the Pharmaceutical Benefit Scheme figure was to ensure that the full patient costs, including pharmacy mark-ups, were included. The assumption was that the Chemist Warehouse prices represented the average market value of these drugs. The market price for these drugs was cheaper, the same or more than the price paid by BLT. They were significantly less if the patient was on a concession card. The quantities of each drug were obtained from BLT records of drugs purchased for the trial of which all were dispensed to trial patients. The proportion of women on the trial with concession cards was unknown, so an SA was used with the concession price as the minimum cost, the normal price as the maximum cost and an average of the two as the midpoint. The repurposed equipment included two Niox Vero machines, two Picos machines and three iPads. These were valued at the price paid by BLT and converted into 2022 dollars. Although all these equipment are currently being used for research and will continue to be used for future research, a conservative approach was taken by simply valuing the cost of the equipment for one subsequent project. The value of an increase in lifetime earnings as a result of PhD completion was estimated at $30 000 per student for two PhD graduates.^21^

**Table 1:** Cost-consequence analysis: costs and monetisable consequences for the Breathing for Life Trial

	Value ($)	Sensitivity analysis ($)	
Costs		**Min**	**Max**
Research costs	2 638 187		
Opportunity costs	116 620		
Implementation costs	71 430	60 407	82 452
Total costs	2 826 237	2 815 215	2 837 259
Consequences			
Funding leveraged	6 780 095	2 869 105	11 476 418
Medication costs saved by patient	28 019	13 606	42 431
Savings in repurposed equipment	15 573	15 573	15 573
Increased lifetime earnings of PhD students	60 000	60 000	60 000
Total consequences	6 883 687	2 958 284	11 594 422

### Narrative

A narrative was constructed using the pathways to impact from the Program Logic Model and supplemented with findings from the Payback analysis and interviews with both clinicians and patients, conducted as part of BLT.^9 10^ In particular, the comments made by patients and clinicians were used ‘verbatim’ to capture and communicate the capacity building and community impacts of BLT.

### Data collection and analysis

The majority of the data used in the BLT Impact Assessment were secondary data available from project administrative records, clinical data collection, chief investigator diaries and curriculum vitae. Where required, costing data were obtained from publicly available or published values. For knowledge advancement, citations for publications were sourced through Scopus and Google Scholar. Full-text downloads were measured using journal websites and the University of Newcastle NOVA database (open-access repository for research outputs of academic staff) where available with a cut-off date of 15 February 2023. In addition to the detailed record keeping and clinical data collected during the study period, qualitative data were also collected as part of a PhD substudy.^9 10^ BLT participants and clinicians were interviewed to obtain their perspectives on BLT. Additional interviews to ascertain the perspective of BLT study staff were also conducted. The interview transcripts were analysed using a secondary content analysis which focused on the different participant/patient, clinician and study team perspectives and experiences of participating in the trial. The aim of the secondary qualitative analysis was to identify the qualitative impact of BLT. The analysis revealed that patients, clinicians and BLT staff were aware of increased awareness and knowledge, changes in clinical practice, as well as improved asthma management as impacts of BLT.

### Patient and public involvement

There was no patient involvement in this research impact assessment. However, the results of this study will be disseminated to study participants via BLT social media, BLT website and BLT participant newsletter.

## Results

### Payback

The retrospective application of FAIT to BLT identified areas of research impact in several domains, including capacity building for clinicians and researchers, knowledge advancement and community benefits such as improvements in quality of care and economic impacts. Of particular significance was the improvement in participants’ asthma management knowledge, including correct asthma plan knowledge, adequate/optimal inhaler technique and correct asthma medication knowledge. Table 2 presents these impact results by various domains of benefit.

**Table 2:** Impact metrics for the BLT study by the Payback domains of benefit

Domains		Metric	Result
Advance knowledge	Published papers (2016–2022)	No. of articles on the BLT project published in peer-reviewed journals, including original research, protocols and editorials	8
	Citations	Average field-weighted citation impact	1.3
		Combined field-weighted citation impact (Scopus)	10.29
		No. of total citations in Scopus	78
	Open access	Percentage of peer-reviewed articles on an open-access platform	37.5%
	Altmetrics	Combined Altmetric score on journal websites	38
	Downloads	No of times articles downloaded on the NOVA database	337
	Downloads	No of times articles downloaded on journal website	479
	Presentations	No. of presentations (total)	80
		No. of international presentations	10
		No. of national presentations	20
		No. of oral presentations	29
	Posters	No. of poster presentations	11
	Media/social media	No. of mentions (paper, radio and television)	7
		No. of Facebook page followers	356
Capacity/capability building	Collaboration—individuals	No. of unique individual collaborators on grants/publications	21
	Collaboration—organisations	No. of unique organisations collaborating on grant/publication	13
	PhDs supervised	No. of PhD students using BLT data in their research	6
	PhDs completed	No. of students completed a PhD funded by BLT	2
	Post docs	No. of post docs employed by BLT	1
	Training in asthma education and relevant testing	No of midwives trained	10
		No. of senior staff specialists trained	4
		No. of nurses and researchers trained	4
Policy	Contribution to policy	No. of CIs on policy forums (European Respiratory Society/Thoracic Society of Australia and New Zealand Task force)	1
		Contribution to the statement on the management of reproduction and pregnancy in women with airways diseases	1
Community benefit	Improvement in asthma planning	No. of participants provided WAAP	508
		Increase in WAAP possession among intervention group participants	From 15% to 100%
	Improvement in the use of asthma management devices (at end of study)	Had adequate or optimal inhaler technique (pMDI)	From 46 to 83%
		Had adequate or optimal inhaler technique (turbuhaler)	From 80% to 97%
		Had adequate or optimal spacer technique	From 22% to 71%
	Improvement in asthma management knowledge (by end of study)	No. of participants given asthma self-management education	1182
		Had correct asthma action plan knowledge	From 12% to 70%
		Had correct reliever medication knowledge	From 25% to 79%
		Had correct preventer medication knowledge	From 23% to 74%
	Improvement in asthma management	Clinical improvement in Asthma Control Questionnaire scores (decrease of 0.5)	268 (45%)
Economic benefits	Medication costs saved	Total value of trial participant medication savings (costed at subsidised hospital pharmacy rates?)	$28 019
	Repurposed equipment	Savings from repurposing of trial equipment	$15 573
	Leveraged grants and fellowships	No. of research grants and fellowships leveraged as a result of BLT	63
		Total value of research grants leveraged by the BLT team	$6.7 million
	Jobs created	Postdoctoral positions created in regional Australia	1
	Increase in lifetime earnings	Increase in lifetime earnings from completed PhDs	$60 000

BLTBreathing for Life TrialWAAPwritten asthma action plans

### Return on investment

The BLT research and implementation cost totalled $2 826 237. This includes both financial and opportunity costs. Taking into account leveraged research and fellowship funding, patient medication savings, savings from repurposed equipment and increased lifetime earnings of PhD graduates funded by BLT, the consequences that were able to be monetised totalled $6 883 687 (SA $2 958 284–11 594 422). Most of the monetisable consequences were in leveraged research and fellowship funding. This translates to a return of $2.44 (SA $1.04–4.12) for every dollar spent on the conduct of BLT from the monetisable consequences. The true value needs to consider the non-monetisable consequences, expressed in table 2. The results of the CCA are presented in table 1.

### Narrative

#### Needs and aims

Controlling asthma in pregnancy can reduce the risk of adverse perinatal outcomes as well as the potential to improve respiratory health outcomes for mothers and their babies. The findings from a previous study demonstrated that using a FeNO-based algorithm to guide asthma therapy reduced exacerbations in pregnancy by 50% among non-smokers.^22^ Therefore, a large-scale RCT was needed to determine the acceptability, effectiveness and the cost of delivering FeNO-guided asthma care compared with usual care. The BLT was set up to test the hypothesis that using a FeNO-based intervention to guide asthma therapy in pregnancy will improve maternal respiratory health, perinatal health and childhood respiratory health outcomes. The trial was conducted over 6 years and incurred $2.8 million in research and implementation costs.

### Impacts

#### Knowledge advancement

In addition to the eight peer-reviewed articles with a total citation account of 78, one paper^23^ was featured in The Limbic and selected for a ‘Latest Research’ highlight on the American Academy of Allergy, Asthma & Immunology website. Another paper^24^ prompted a response to the editor which highlighted how BLT represents “*a significant contribution to the literature by expanding the evidence on medication non-adherence in pregnancy and providing a framework for clinicians to improve the quality of asthma care*.”^25^ On three occasions, VEM was invited to the Perinatal Society of Australia and New Zealand’s Clinical Trials Network (IMPACT: Interdisciplinary maternal and perinatal Australasian clinical trials network) to present on BLT to build the capacity of perinatal researchers across Australia and New Zealand.

Another contribution to both knowledge advancement and capacity building was the development of an asthma in pregnancy online toolkit that was partly informed by findings from BLT. The toolkit is a ‘Best Practice’ go-to information resource for Asthma in Pregnancy care for healthcare professionals and community members that has been endorsed by Asthma Australia and the Society of Obstetric Medicine of Australia and New Zealand. There are eight BLT papers cited in the toolkit, as well as six papers from BLT follow-up studies. In its first 8 months, it has been accessed by over 13 000 unique users from 73 countries worldwide.^26^ A BLT Facebook page was created in 2016 and has 356 followers, with the most engaged post reaching 822 Facebook users.

#### Capacity building

Academics, clinicians, allied health and research staff who participated in BLT represented 12 different national and international universities, health and research-based institutions. The collaboration with Canberra Hospital was a particularly beneficial partnership as it led to further collaborations and the successful award of an asthma in pregnancy health promotion campaign grant in 2022.

Staff from the study sites were invited to participate in capacity-building opportunities, including research and clinical skills training sessions/workshops. Educational resources, including training manuals and instructional videos, were developed and used as part of this process. Training covered respiratory testing and measurement, including how to conduct spirometry (used to assess lung function by measuring inhalation and exhalation), exhaled carbon monoxide measurement and FeNO testing, which provides a way of measuring eosinophilic airway inflammation. Staff training also included education around asthma in pregnancy, how to check and correct asthma inhaler technique, assessment of medication use and adherence, participant recruitment and general research protocol knowledge, where a training manual was provided to each study site.

#### Community benefits

Secondary analysis of BLT data revealed that there were significant improvements with the intervention group in areas of asthma knowledge, quality of care and asthma control. Overall, 1182 trial participants were given asthma self-management education which included demonstrated correct device technique for inhalers and spacer over 1800 times. 508 participants were provided written asthma action plans. Improvements in knowledge and asthma management and knowledge were recorded with a 76 percentage point increase in written action plan possession and a 58 percentage point increase in correct written action plan knowledge. Written action plan knowledge was defined as participants being able to verbalise the steps of their action plan such as knowing what to do when their symptoms worsen. There were also marked improvements in asthma symptoms measured by Asthma Control Questionnaire (ACQ) scores, with 44% of participants having clinically improved asthma (ACQ reduction of at least 0.5), while 41% of participants had no change in asthma control.

Staff were interviewed about their general involvement in the study as well as the impact of the training on their practice. When describing how their clinical practice has changed since participating in BLT, one staff member said:

I would be asking them what medication - what they use to treat their asthma … I'd certainly be encouraging them to not stop their medication and, obviously now I talk very differently about it and how to use their medication and I wouldn't have done that before.

Another described the improvements in asthma knowledge over the course of the study:

I found that they actually didn't know stuff, they didn't know that asthma and a lack of oxygen could affect the baby. They didn't realize that if they felt bad it could potentially impact baby, in terms of, you know, inter uterine growth restriction or premature birth. They didn't know anything about it so it was nice to actually kind of get feedback from them saying “I didn't know anything about this. I didn't realize my baby could be potentially affected if my episode control.” So a lot of little things they got out of it apart from just a monetary benefit (free medication).

Clinical staff remarked on the clinical improvements that they observed with patients who participated in BLT:

I've seen the benefit of women who will then have their medication changed and come back the next appointment and go, wow, that’s what it’s like to breathe normally. That’s what I like about this study because you get that feedback from women.

These improvements in knowledge and clinical indicators were confirmed during participant interviews, where several described their learning from participation in the study:

… it’s taught me a lot more as well in regards to the baby and how it [asthma] affects the baby. (PW5JHH)

I had absolutely no idea that it could even change in pregnancy …. Very eye opening because obviously if I am starved of oxygen then so is my baby. (PW11JHH)

The most frequent comment that participants made about the benefits of participating in the study was regarding improvements in quality of care and asthma control.

[Kelly] (Study staff) has put me on a different preventer – Symbicort and it is honestly completely changed my asthma so I can exercise and what not and I don’t even have to have my Ventolin. It is amazing. I can’t believe the Drs have not put me on it before. (#18JHH)

I was part of the monthly one where they did lots of testing and Kelly made sure that I was on the right dosage of medication for what I needed. If I had an exacerbation, they jumped right onto that. They did all the testing to see where I was sitting every single time so that they had a really good idea of what my asthma was doing. (PW1JHH)

… A lot of pregnant women don’t like being on medications when they’re pregnant. Yeah, it just adds to my medication, so it’s - yeah unfortunately. It’s something I have to live on, but yeah it is. It’s good because you’re not overdosing yourself either. (PW8JHH)

## Discussion

### Novelty of the study and the principal findings

This is the first time FAIT has been applied to a clinical trial, and despite being a negative trial, FAIT application was able to show that there were many positive consequences and impacts of the BLT, which included improved clinician and patient knowledge, building of research capacity, improved patient care and a $2.44 return on investment for every dollar spent. Despite being a retrospective application, detailed record keeping and existing qualitative data available through a BLT-funded PhD enabled us to evidence the many useful impacts outside of traditional academic publications.

### Strengths and limitations

A key strength of the FAIT application was the combination of three existing validated methods for assessing the impact of research, including quantitative impact metrics grouped by domains of benefit, a CCA that captures the return on research investment and a narrative account of the impact through the voices of beneficiaries of the research. This comprehensive assessment methodology ensured a broad perspective was taken with the research impact assessment and that the results have the potential to appeal to a wider audience, including researchers, policymakers and research funders.

There were limitations with the retrospective application of the FAIT framework, associated with reliance on existing research and administrative records. This necessitated a conservative approach, and other impacts could potentially have been captured had an impact assessment been planned from the beginning. The limited funding also meant no opportunity for primary data collection that would have potentially evidenced additional benefits not covered by this application. For example, the value to clinicians (midwives, obstetricians and general practitioners) of being involved in the BLT and their understanding of the importance of better asthma management in pregnancy and potential changes to their future practice.

### Key learnings

Even though this impact assessment was applied retrospectively, detailed record keeping of all trial activities, including training, patient visits, evaluation and general grant administration, provided the evidence needed to apply all three FAIT methods to BLT. A key take-home message for all health medical research is the need to keep detailed records of all research activities in case the need for an impact assessment arises. A second take-home message is that clinical trials, even when the findings reject the hypothesis, have the potential to have positive impacts not just on future research, but on building research capacity, saving money on future research through repurposing technical equipment, improving the health knowledge of consumers involved in the research and potentially improving the healthcare they receive and their health outcomes in the future. This last point has been previously covered in the literature.^27^

### Implications for researchers and policymakers

This study demonstrates the proof of concept for the conduct of a retrospective impact assessment of a clinical trial. The investment in clinical trials is often substantial from a monetary perspective, and considering a trial a success based only on the primary outcome measure is narrow and misses the opportunity to understand the broader benefits of clinical trials. Spin-off benefits of the trial can include improving knowledge, capacity, service delivery models and can have serendipitous outcomes even for the community through participation in the trial. The inclusion of research impact plans in funding guidelines may be a useful addition to help researchers design clinical trials with more opportunities for benefit, other than the primary outcome of the trial.

This trial highlighted important deficits in asthma care, including a lack of written asthma action plans among pregnant women, providing evidence that guidelines are not being followed. Alternate methods in addition to guidelines are required to ensure best practice healthcare is being delivered.

Pregnancy is an important and unique time period when the health of the mother can impact the future health of her child, providing an important window of opportunity for intervention. In the case of asthma, having a mother with asthma is the single biggest risk factor for childhood asthma, greater than having a father with asthma,^28^ and exacerbations or uncontrolled asthma during pregnancy further increase the risk of asthma in the offspring.^29^ In BLT, much of the leveraged funding enabled follow-up studies of the children born to BLT mothers, including studies related to lung health^30^ and the developing immune system,^31^ studies of nutrition^32^ and body composition, and studies of neurodevelopment.^33^,^35^

## Conclusion

The application of FAIT to retrospectively assess the impact of BLT was a viable and beneficial way of identifying the research impact of a negative clinical trial. Although the intervention in the trial did not improve the intended primary outcomes measures for mothers and babies, FAIT identified other substantial impacts of the study. In order to ascertain the wider-reaching impacts of health and medical research, future studies should consider incorporating FAIT prospectively to gain a more holistic measure of research impact.

## Data Availability

Data are available upon reasonable request.
