# Supplementary material for: What are the broader impacts and value from a randomised controlled trial conducted in six public hospital antenatal clinics in Australia? An impact assessment using the Framework to Assess the Impact from Translational health research
Source: BMJ Open. 2025 Mar 26;15(3):e082795. doi: 10.1136/bmjopen-2023-082795 (PMC11950953; doi:10.1136/bmjopen-2023-082795)
Supplement: Supplementary file 1 [file bmjopen-2023-082795.reviewer_comments.pdf]

PEER REVIEW HISTORY

BMJ Open publishes all reviews undertaken for accepted manuscripts. Reviewers are asked to complete a checklist review form and are provided with free text boxes to elaborate on their assessment. These free text comments are reproduced below.

ARTICLE DETAILS

Title (Provisional)

What are the broader impacts and value from a randomised controlled trial conducted in six public hospital antenatal clinics in Australia? An impact assessment using the Framework to Assess the Impact from Translational health research

Authors

Elvidge, Elissa; Murphy, V; Rao, M; Gibson, Peter G; McLauchlin, Karen; Robijn, Annelies; Jensen, Megan E.; Callaway, Leonie Kaye; Attia, John; Hensley, Michael; Giles, Warwick; Peek, Michael; Barrett, Helen; Seeho, Sean; Mattes, Joerg; Abbott, Alistair; Bisits, Andrew; McCaffery, Kirsten; Colditz, Paul B; Searles, Andrew; Ramanathan, Shanthi Ann

---

VERSION 1 - REVIEW

**Reviewer**	**1**
**Name**	**Ali, Mohammad**
**Affiliation**	**Mohammed Al-Mana College for Medical Sciences, Pharmacy**
**Date**	**20-Jan-2024**
**COI**	**None.**

It is an honour for me to review manuscript number "bmjopen-2023-082795" titled " More than meets the eye: taking a broader assessment of the impact and value of the Breathing for Life randomised controlled trial " for the "BMJ'' journal. The manuscript looks interesting however, the following major modification is required.

• The introduction is poorly described and not aligned with the study objective

• Kindly add the rationale of the study under the last paragraph of the introduction

• Kindly check the citation style writing in the entire manuscript unify and correct whenever applicable such as page 11 line number 54.

• Kindly write the study duration under the methodology

• How follow-up was performed for enrolled participants

• Kindly add study Ethical approval number under methodology

• How respondents were identified

• Kindly add the study site

• How study respondents biased were minimized

• Kindly add exclusion criteria for the study

• Discussion needs to update and compare findings with the latest published article

• Kindly add limitations of the study

• Please scan your manuscript again and correct some topological errors

• Please make sure your manuscript was written in line with this journal submission guideline

---

**Reviewer**	**2**
**Name**	**Asamoah-Boaheng, Michael**
**Affiliation**	**Memorial University of Newfoundland**
**Date**	**14-Feb-2024**
**COI**	**No competing interest**

The researchers retrospectively assessed and reported on the impact and and value of BLT to show accountability of research investment.

The paper is well written and I commend the authors for a great job done. Kindly see the following minor comments for your attention:

Ethics approval: Although the researchers used secondary data for this study, I would recommend they make a statement in the manuscript indicating that "ethics application/approval for this study was not necessary/needed". Or if they received ethics approval, they should try and mention it in the manuscript.

Results

Also, I was expecting to at least see a table that shows descriptive characteristics of the individuals who participated in the study.

-->Also, a pictorial/flow chart description of the FAIT concept in the manuscript would be beneficial for readers to understand and appreciate it more.

**Reviewer**	**3**
**Name**	**Graves, Nicholas**
**Affiliation**	**Queensland University of Technology**
**Date**	**24-Sep-2024**
**COI**	**No COIs.**

This is an interesting paper and I appreciate the chance to provide some comments.

My feeling is that the authors are being defensive about their trial. It reads like the authors want to justify the costs of the trial, with some quantified economic returns, just because it was a negative finding. Negative findings are important and should be disseminated the same as positive findings. Having evidence as to why we should not do something is just as important as the opposing situation. A major threat to the credibility of the research community is that we only publish "good news" from our research, yet many studies can't be reproduced. The tone of the paper might be changed. Tell the reader what a great trial was done and that you got a negative finding. If your funders and peers are critical of negative trial results, then they need to improve their understanding of what research is.

The work you have done on payback from this research is potentially useful, but you need to clearly separate this work from the trial findings, which will stand alone.

The authors used the Framework to Assess the Impact of Translational health research to structure an evaluation of the research payback, economic return on investment, and, they write a narrative of the impact. Evidence synthesis was used to generate results.

This framework is not a golden bullet as implied by the authors. Rather it draws on standard approaches to impact evaluation. The mainstay is to use a logic model and there is nothing novel about that. I suggest the authors tone down the superlatives for the framework. The evaluation is only as good as the data assembled. The framework used is merely a guide for the research team.

Figure 1 is not a figure it's a table. It might be a good idea to draw a picture, which is a figure, and then put the test for the figure in an online appendix. Please remove the acronyms. It makes the paper harder to read as you are using code words that no one

understands. Readers must search your paper for the key to your code. When I read “The PLM was developed by the BLT team” I thought I was going to get a sandwich.

For the ‘Costs’ section on page 9, I think you can report the results better. I suggest you report in money terms ($) only the costs that changed because the trial was undertaken. These might include expenditures on variable cost items. Think what money/cash could have been put in a bag and given to someone else for some other valuable activity if the trial had not happened. These estimates could include staff who were employed specifically to run the trial. There will be some sunk or fixed costs, such as staff who allocated their valuable time to the trial. For this I would aim to quantify how much time was provided and then, as a secondary step, report the value of that time by generic remuneration rates. For example, a medical consultant spent 50 days on the trial; and the money required to make them come to work for a day is $2,000. If the trial was not run then $100,000 would not be saved in cash, rather 50 days would be available for the medical consultant to do something else.

For the consequences section I think the authors have done a decent job of estimation. There is a lot of uncertainty about the real economic gains from items 1 to 4 listed, and I sense the authors appreciated this.

Might the author make the assumptions used for the Table 2 calculations open access. You might publish the spreadsheets used for the calculations, so others can see the methods you used.

I though the narrative results were good. These describe the richness of the activity associated with the trial and its subsequent value. I’m not a qualitative researcher but the qualitative data included were informative.

Overall, I think this is a useful piece of work - congratulations. BMJ OPEN is a good journal for this paper.

VERSION 1 - AUTHOR RESPONSE

**Reviewer 1:** Dr. Mohammad Ali, Mohammed Al-Mana College for Medical Sciences<br>Comments to the Author: It is an honour for me to review manuscript number "bmjopen-2023-082795" titled " More than meets the eye: taking a broader assessment of the impact and value of the Breathing for Life randomised controlled trial " for the "BMJ'' journal. The manuscript looks interesting however, the following major modification is required.		
**Reviewer comments**	**Response from authors**	**Changes to the manuscript**
The introduction is poorly described and not aligned with the study objective	Thank you for this feedback. Re-reading this section we have realised that we needed to describe the context of this assessment first and have now done so. Information about the BLT trial is introduced later and the level of information reduced. .	**New introduction:**<br>Every year Australia spends $10 billion on health and medical research which is approximately 4% of all spending on health (total health expenditure in 2021-22 was $241 billion) (1) Of this expenditure, approximately 10 per cent or $1.1 billion is spent on clinical trials activity (2) . Clinical trials are important. They provide essential evidence for more effective and lifesaving therapies and identify ineffective and unnecessary interventions. They also help find ways to detect diseases early, when they are more treatable. Clinical trials advance medical knowledge, which leads to better health outcomes for patients and hospitals that conduct clinical trials tend to provide better care and have lower mortality rates. (3)<br><br>Despite this large investment of public monies and the global benefits that clinical trials can bring, very little is known about the benefits that result from individual clinical trials, especially when they have a negative result. With the impact of COVID and the rising cost of healthcare, there is an added imperative for health and medical researchers to be accountable for the spending of public monies. In Australia and globally there is a growing demand for more accountability in public spending across all sectors, including health and medical research. [15] Despite this, impact assessment beyond academic outputs such as peer-reviewed publication citations or field weighted citation indexes is still not standard practice in many countries. [16]. The value of negative trials is often underestimated in terms of the new knowledge and benefits they generate over and above an often necessarily narrow set of outcomes to the exclusion of other potentially meaningful outcomes.
Kindly add the rationale of the study under the last paragraph of the introduction	It was partially there but we have added the objective	Given the significant investment of public funds in this "negative" trial, the BLT research team were keen to be as transparent and accountable as possible for the investment and to assess the impact and value of BLT using a broader societal lens. With support from the Health Economics and Impact team at the Hunter Medical Research Institute, the research team undertook a retrospective impact assessment of the

		Breathing for Life Trial. The objective was to assess and evidence the broader impacts and benefits from the trial and give visibility to its value and return on the research investment.
Kindly check the citation style writing in the entire manuscript unify and correct whenever applicable such as page 11 line number 54	Thank you for this feedback	The citation styles have been checked and corrected throughout.
• Kindly write the study duration under the methodology<br>• How follow-up was performed for enrolled participants<br>• Kindly add study Ethical approval number under methodology<br>• How respondents were identified<br>• Kindly add the study site<br>• How study respondents biased were minimized<br>• Kindly add exclusion criteria for the study	This set of questions are appropriate and necessary when applied to the original clinical trial.<br><br>This manuscript reports on the retrospective application of an impact assessment methodology to a completed and published clinical trial to assess its broader benefits. None of these requests are relevant to this study.	No changes made
Discussion needs to update and compare findings with the latest published article	There are no previous applications of FAIT or any other research impact assessment to a randomised control trial. That is the novelty of this manuscript.	No changes made
Kindly add limitations of the study	Limitations were included but have been	Please refer to comments in a previous section regarding the Limitations

	further expanded.	
• Please scan your manuscript again and correct some topological errors<br>• Please make sure your manuscript was written in line with this journal submission guideline	Apologies for all typographical errors.<br>We have reviewed the guidelines and headings have been added or renamed and required sections added	All typographical errors have been corrected. Please refer to previous comments re multiple changes made

**Reviewer 2**: Dr. Michael Asamoah-Boaheng, Memorial University of Newfoundland<br>Comments to the Author: The researchers retrospectively assessed and reported on the impact and value of BLT to show accountability of research investment. The paper is well written and I commend the authors for a great job done. Kindly see the following minor comments for your attention.		
**Reviewer comments**	**Response from authors**	**Changes to the manuscript**
Ethics approval: Although the researchers used secondary data for this study, I would recommend they make a statement in the manuscript indicating that "ethics application/approval for this study was not necessary/needed". Or if they received ethics approval, they should try and mention it in the manuscript.	Previously covered. Information has been added as requested.	**Ethics approval**<br>Ethics approval was not required for this study that relied solely on the administrative records and previously collected data from the trial which was covered by the following ethics approvals: Hunter New England Local Health District Human Research Ethics Committee (Reference number 12/10/17/3.04) and the Australian Capital Territory Health Human Research Ethics Committee (ETH.11.15.232).
Results- Also, I was expecting to at least see a table that shows descriptive characteristics of the individuals who participated in the study.	There were no participants in this research impact assessment. The analysis was based on administrative records and previously collected feedback.	No change made.
Also, a pictorial/flow chart description of the FAIT concept in the manuscript would be beneficial for readers to understand and appreciate it more.	This is a great suggestion to improve reader understanding. We have added a pictorial.	Figure 1: Framework to Assess the Impact of Translational health research (FAIT)

**Reviewer 3**: Dr. Nicholas Graves, Queensland University of Technology<br>Comments to the Author: This is an interesting paper and I appreciate the chance to provide some comments.		
**Reviewer comments**	**Response from authors**	**Changes to the manuscript**
My feeling is that the authors are being defensive about their trial. It reads like the authors want to justify the costs of the trial, with some quantified economic returns, just because it was a negative finding. Negative findings are important and should be disseminated the same as positive findings. Having evidence as to why we should not do something is just as important as the opposing situation. A major threat to the credibility of the research community is that we only publish "good news" from our research, yet many studies can't be reproduced.<br>The tone of the paper might be changed. Tell the reader what a great trial was done and that you got a negative finding. If your funders and peers are critical of negative trial results, then they need to improve their understanding of what research is.	We agree wholeheartedly with the views of this reviewer and the results of the BLT clinical trial have been extensively published as reported in the introduction (add some references). This paper was not an attempt to justify the trial but to add a broader perspective on the outcomes. We hope the new introduction that describes the context for this study will make that clearer.	Changes to the Introduction as previously described.
The work you have done on payback from this research is potentially useful, but you need to clearly separate this work from the trial findings, which will stand alone.	The Payback metrics are a combination of data from various administrative and previously published sources. The findings are not provided as part of this analyses.	No changes made
The authors used the Framework to Assess the Impact of Translational health research to structure an evaluation of the research payback, economic return on investment, and they write a narrative of the impact. Evidence synthesis was used to generate results.<br><br>This framework is not a golden bullet as implied by the authors. Rather it draws on standard	Overall, we believe FAIT is an appropriate framework that provides a comprehensive and multi-dimensional assessment of the impact of the BLT study. It has enabled the presentation of knowledge, research capability and capacity building, research infrastructure, policy and economic impacts, an estimate of the return on the research investment and the	Some superlatives removed.

approaches to impact evaluation. The mainstay is to use a logic model and there is nothing novel about that. I suggest the authors tone down the superlatives for the framework. The evaluation is only as good as the data assembled. The framework used is merely a guide for the research team.	voices of BLT study investigators and consumers describing the broader impact of the study. Yes we agree it is not a magic bullet and is only as good as the evidence we were able to provide. We have removed unnecessary superlatives but maintain our evaluation of its value and comprehensiveness.	
Figure 1 is not a figure it's a table. It might be a good idea to draw a picture, which is a figure, and then put the test for the figure in an online appendix.	This is now Figure 2 and has been created as an image.	New file provided for insertion
Please remove the acronyms. It makes the paper harder to read as you are using code words that no one understands. Readers must search your paper for the key to your code. When I read "The PLM was developed by the BLT team" I thought I was going to get a sandwich.	Thank you for the laugh. We have removed as many acronyms as possible throughout the paper.	All reference to PLMs have been changed to Program Logic Model. As far as practicable, all references to BLT have been changed although some have been left in for brevity.
For the 'Costs' section on page 9, I think you can report the results better. I suggest you report in money terms ($) only the costs that changed because the trial was undertaken. These might include expenditures on variable cost items. Think what money/cash could have been put in a bag and given to someone else for some other valuable activity if the trial had not happened. These estimates could include staff who were employed specifically to run the trial. There will be some sunk or fixed costs, such as staff who allocated their valuable time to the trial. For this I would aim to quantify how much time was provided and then, as a secondary step, report the value of that time by generic remuneration rates. For example, a medical consultant	The methodology for the cost-consequence details out exactly what you have described. We have separated the "costs" into financial costs (what you call money terms) and opportunity costs (in terms of in-kind contributions to the project activity that may not have been renumerated but could have been allocated to another activity). These are covered in detail the methods section and in the calculations that informed the cost-consequence analysis. We have added a sentence in the results with reference to this.	**Added sentence:**<br>This includes both financial and opportunity costs.

spent 50 days on the trial; and the money required to make them come to work for a day is $2,000. If the trial was not run then $100,000 would not be saved in cash, rather 50 days would be available for the medical consultant to do something else.		
For the consequences section I think the authors have done a decent job of estimation. There is a lot of uncertainty about the real economic gains from items 1 to 4 listed, and I sense the authors appreciated this.<br><br>Might the author make the assumptions used for the Table 2 calculations open access. You might publish the spreadsheets used for the calculations, so others can see the methods you used.	Thank you. Unfortunately the spreadsheets we use for the calculations are not able to be shared but we would be happy to share more details of the methods we used with individual readers who would like to engage with us.	No changes made
I thought the narrative results were good. These describe the richness of the activity associated with the trial and its subsequent value. I'm not a qualitative researcher but the qualitative data included were informative.	Thank you.	No changes required
Overall, I think this is a useful piece of work - congratulations. BMJ OPEN is a good journal for this paper.	Thank you for your positive encouragement	No changes required

VERSION 2 - REVIEW

Reviewer 3
Name Graves, Nicholas
Affiliation Queensland University of Technology
Date 06-Feb-2025
COI

All good, thanks for addressing the comments
